# Changes in Medical Management after Coronary CT Angiography

**DOI:** 10.5935/abc.20150088

**Published:** 2015-10

**Authors:** Vânia Mairi Naue, Gabriel Camargo, Letícia Roberto Sabioni, Ronaldo de Souza Leão Lima, Maria Eduarda Derenne, Andréa Rocha de Lorenzo, Monica Di Calafiori Freire, Clério Francisco Azevedo Filho, Elmiro Santos Resende, Ilan Gottlieb

**Affiliations:** CDPI - Clínica de Diagnóstico por Imagem

**Keywords:** Coronary Artery Disease, Diagnostic Imaging, Atherosclerosis/physiopathology, Therapeutics

## Abstract

**Introduction:**

Coronary computed tomography angiography (CCTA) allows for non-invasive coronary artery disease (CAD) phenotyping. There are still some uncertainties regarding the impact this knowledge has on the clinical care of patients.

**Objective:**

To determine whether CAD phenotyping by CCTA influences clinical decision making by the prescription of cardiovascular drugs and their impact on non-LDL cholesterol (NLDLC) levels.

**Methods:**

We analysed consecutive patients from 2008 to 2011 submitted to CCTA without previous diagnosis of CAD that had two serial measures of NLDLC, one up to 3 months before CCTA and the second from 3 to 6 months after.

**Results:**

A total of 97 patients were included, of which 69% were men, mean age 64 ± 12 years. CCTA revealed that 18 (18%) patients had no CAD, 38 (39%) had non-obstructive (< 50%) lesions and 41 (42%) had at least one obstructive ≥ 50% lesion. NLDLC was similar at baseline between the grups (138 ± 52 mg/dL vs. 135 ± 42 mg/dL vs. 131 ± 44 mg/dL, respectively, p = 0.32). We found significative reduction in NLDLC among patients with obstrctive lesions (-18%, p = 0.001). We also found a positive relationship between clinical treatment intensification with aspirin and cholesterol reducing drugs and the severity of CAD.

**Conclusion:**

Our data suggest that CCTA results were used for cardiovascular clinical treatment titration, with especial intensification seen in patients with obstructive ≥50% CAD.

## Introduction

Cardiovascular clinical treatment titration in patients without prior diagnosis of coronary artery disease (CAD) is based on patient cardiovascular risk estimated by clinical variables, being generally indicated in patients classified as high risk^1^.

Coronary computed tomography angiography (CCTA) is generally used with high accuracy^2^ for obstructive CAD diagnosis and, as it allows three-dimensional evaluation of the wall vessel, it also provides non-obstructive CAD visualization, showing good correlation with intravascular ultrasound^3^.

The main therapeutic intervention used in patients with atherosclerosis, as a means of primary prevention of ischemic events, are Cholesterol-Lowering Drugs (CLD)^4^. However, the number needed to treat (NNT) varies according to the studied population and low cardiovascular risk patients benefit less than those at high cardiovascular risk. However, the potential for adverse effects remains similar^1,5,6^. According to the variation of baseline risk, the NNT may vary from 24 to 549 treated patients, for the reduction of an event^5^.

Studies show that approximately 20% to 30% of asymptomatic patients considered at low cardiovascular risk (event rate less than 10% in ten years) have atherosclerosis in the coronary computed tomography angiography (CCTA)^7,8^ and it is known that these findings are associated with increased incidence of cardiovascular events, independently from and in addition to the clinical risk factors^9,10^. However, it is still uncertain how doctors use the results of CCTA in the clinical treatment titration of their patients.

This study aimed to evaluate the changes in both the prescription and plasma cholesterol levels in the short term, after a CCTA assessment in patients with no prior diagnosis of CAD, according to the severity of CAD found at the examination.

## Methods

A retrospective and analytical cohort was analyzed, and the project was approved by the Research Ethics Committee of HUCFF/FM/UFRJ, under protocol number 27341114.7.0000.5257.

All patients (123) submitted to CCTA between the years 2008 and 2011 in a cardiac imaging laboratory in Rio de Janeiro, with no prior diagnosis of CAD (i.e. without myocardial revascularization history or AMI and no previous CCTA) and had cholesterol measurement recorded at two different times: one up to three months before the CCTA (index measurement), followed by a second sample taken from three to six months after the CCTA (follow-up measurement) were included in this analysis. This period was chosen due to the homogeneity of image acquisition protocols used at that time.

In this institution, a physician from the team performs an interview with the patient prior to the examination, in which information such as anthropometric data, indication for the examination, risk factors, current medications and previous examinations is recorded. Access to the existing clinic database was requested for this study.

The analyzed items were: gender, age, CCTA indication (asymptomatic, typical pain, atypical pain or dyspnea), risk factors (hypertension, diabetes, dyslipidemia, sedentary lifestyle, smoking and family history), medications being used in the index consultation and at the second consultation, such as antiplatelet drugs and Cholesterol-Lowering Drugs (CLD) and the cholesterol levels both at the index and the second consultations. CLD were defined as any drug of the statins or fibrates classes.

Patients whose medical records did not provide the necessary data for this analysis, such as current medication or present risk factors were excluded from the analysis. Additionally patients whose imaging tests had inadequate quality for analysis in three or more coronary segments were also excluded.

The primary outcome of this study was non-LDL cholesterol (NLDLC) reduction after assessment by CCTA in the pre-specified period (three to six months). The NLDLC was considered as the sum of VLDL cholesterol and LDL cholesterol. It was decided to restrict the follow-up to such short period in order to minimize the influence of factors rather than the CCTA outcome on therapeutic decision-making.

As a secondary outcome, the change in medications prescription after the CCTA outcome. was assessed.

CCTA images were acquired using 256-channel devices (BrillianceTIC, Philips Healthcare®, Cleveland, Ohio) or one of the two 64-channel scanners (Brilliance64, Philips Healthcare®, Cleveland, Ohio, SomatomSensation 64, Siemens Healthcare®, Erlangen). For CCTA acquisition, venous and oral beta-blockers were used aiming to reduce HR to less than 60 bpm. Isosorbide dinitrate, 0.4 mg, sublingually was also administered to all patients without contraindications, 3 to 5 minutes prior to image acquisition.

Image analysis was performed by a single expert that had broad experience with the method. The coronary plaques were defined as the presence of image with soft tissue density ≥ 1 mm^2^ compatible with coronary atheromatosis, whereas the degree of luminal stenosis was defined as the ratio between the smallest luminal diameter at the lesion and the reference diameter closest to the lesion.

Patients were classified according to the highest degree of coronary stenosis found, considering: I - no plaque; II - non-obstructive plaques only (< 50% in stenosis); III - at least one obstructive plaque (≥ 50% in stenosis). To measure the cholesterol-lowering effects, a positive CCTA was considered the one with any evidence of coronary atherosclerosis.

### Statistical analysis

The following software programs were used for data processing: SPSS version 19.0 and Microsoft Excel 2000© (9.0.2812).

To calculate sample size, we considered a difference of 30% in LDL cholesterol between patients with positive (estimated at 30% of the sample) and negative CCTA (70% of the sample). The estimated "n" was 90 patients for an alpha error of 0.05 and beta of 0.25.

Quantitative data showed normal distribution through the Kolmogorov-Smirnov test with a significance level of 5%. Continuous variables were expressed as mean ± standard deviation and were compared using paired and unpaired Student’s *t* test, as appropriate. Categorical variables were expressed as amounts and proportions and were compared using the Chi-square and Fisher tests, when appropriate. p values <0.05 were considered significant.

Patients were divided into three groups: without CAD, non-obstructive CAD (< 50% in stenosis) and obstructive CAD (≥ 50% in stenosis). Statistical analysis was carried out for each group separately and the two periods were compared by paired-t test.

## Results

Of a total of 123 patients that had two cholesterol measurements recorded in the proposed time period, 24 were excluded due to incomplete filling out of medical records and two due to poor quality of the images, resulting in 97 patients included in the analysis, of which 67 (69%) were men. The mean age was 64 ± 12 years. Nineteen percent had no risk factor; 15% had only one risk factor; 35% had two or three risk factors; and 31% had four or more risk factors for CAD.

CCTA was performed to assess pain with angina characteristics in 10% of patients; atypical pain in 16%; dyspnea or decreased functional capacity of 23%; and 51% of patients were asymptomatic. The clinical characteristics are described in Table 1. CCTA was normal in 18 (18%) patients, showed no obstructive CAD < 50% in 38 (39%) patients and obstructive CAD ≥ 50% in 42 (43%) patients.

**Table 1:** Population characteristics of individuals submitted to CCTA

**Variables**		
Age (years)		64.2 ± 12
**Gender**		
	Male	67(69)
	Female	30(31)
**Risk Factors**		
	Hypertension	57(58)
	DM	23(24)
	DLP	57(58)
	Family history	35(36)
	Smoking	13(13)
	Sedentary life style	17(17)
**Indications of CCTA**		
	Without pain	50 (51)
	Typical angina	10(10)
	Atypical angina	16(16)
	Dyspnea and exercise intolerance	11(11)

DM: diabetes mellitus; DLP: dyslipidemia; CCTA: Coronary computed tomography angiography

### Cholesterol levels

**Chart 1:**
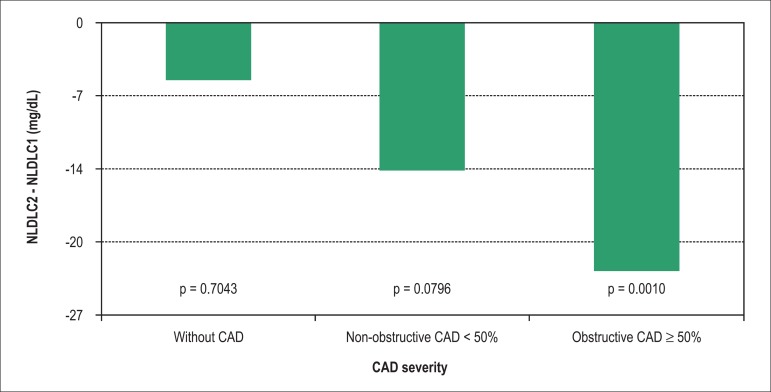
Difference of NLDLC levels pre and post-CCTA when divided into categories according to CAD severity (without CAD, lesions < 50% and lesions ≥ 50%). CAD: coronary artery disease; NLDLC: non-LDL cholesterol.

NLDLC significantly decreased from 136 ± 44 mg/dL in the first to 117 ± 38 mg/dL in the follow-up visit (p = 0.007), a 14% decrease in the average of the general population (Chart 1).

Between the index and the follow-up visit, the variation in NLDLC in the group with negative CCTA was 4% (137 ± 53 mg/dL to 132 ± 39 mg/dL, p = 0.7), in the group with non-obstructive CCTA was 10% (135 ± 42 mg/dL to 121 ± 39 mg/dL, p = 0.07) and the in group in which CCTA showed obstructive lesions was 18% (130 ± 44 mg/dL to 107 ± 36 mg/dL, p = 0.001). There was no significant difference in NLDLC values between the different groups of CCTA in the index visit (p = 0.3).

**Chart 2:**
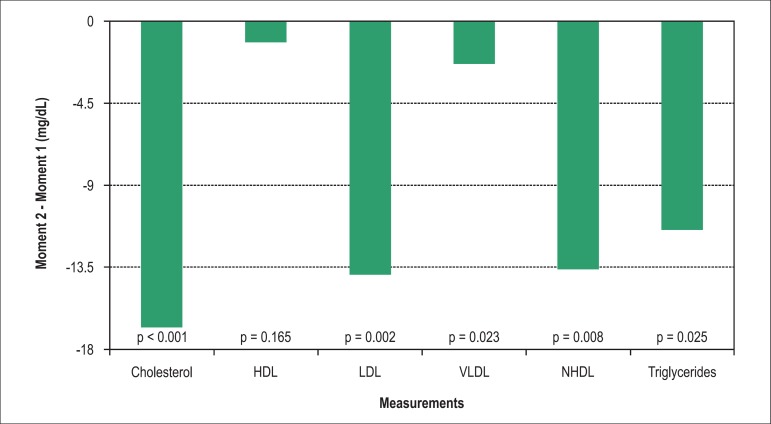
Changes in the decrease of total cholesterol levels, its fractions and triglycerides, pre and post-CCTA. NLDLC: non-LDL cholesterol; HDL: high-density lipoprotein; NHDL: non-high-density lipoprotein; LDL: low-density lipoprotein; VLDL: very low-density lipoprotein.

Of the 42 individuals that had lesions ≥ 50% in stenosis, 32 (76%) showed LDL values < 100 mg/dL at the second visit (Chart 2).

### Medication use

**Chart 3:**
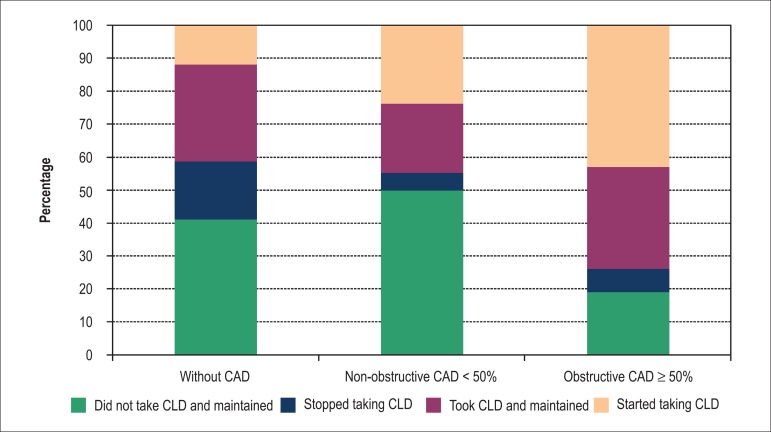
Changes in therapy with cholesterol-lowering drugs, according to CAD severity, demonstrated by CCTA (without CAD, lesions < 50% and lesions ≥ 50%). CAD: coronary artery disease; CLD: chodesterol lowering drugs.

Considering only the 64 (65%) patients that did not use the CLD in the index visit, 28 (43%) started using them after CCTA, when the latter showed at least one coronary segment with lesion (obstructive or not) *versus* 2 (3%) patients with normal CCTA (p < 0.05). Considering only the 34 patients that used CLD in the index visit, 8 (23%) had negative CCTA (of which 3 discontinued use of CLD) and 5 had at least one coronary segment with lesion (obstructive or not) and discontinued these drugs. Chart 3 illustrates the dynamics between treatment with CLD and CCTA results.

**Chart 4:**
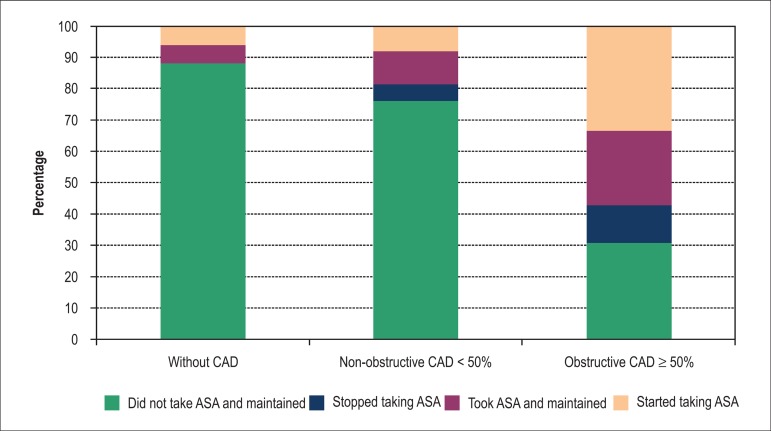
Aspirin prescription as primary preventive therapy, pre and post-CCTA. CAD: Coronary artery disease; ASA: aspirim.

Aspirin use was started after the normal CCTA result in 2 (11%) patients and in 17 (21%) patients with positive CCTA (Chart 4). Importantly, 15 (36%) patients with obstructive lesions were not using any antiplatelet agents on the second visit.

The combined use of aspirin and CLD was started at 0%, 2% and 19% of patients with negative CCTA, with non-obstructive lesions and lesions ≥ 50%, respectively (p = 0.004), and only one of the two drugs alone in 18%, 24% and 38% respectively (p = 0.006).

## Discussion

CAD phenotyping by CCTA has a relevant prognostic impact and improves risk classification for cardiovascular events when compared to the classic risk factors^11-14^.

This study allowed to evaluate, in the real world and in the short term, the impact of CCTA results on drug therapy, evaluating trends in cholesterol levels and the use of medications shortly after the CCTA results.

The analysis of this impact on clinical therapy is relevant, as the benefit of antiplatelet agents and CLD is directly associated with the patient’s cardiovascular risk. It has been demonstrated that in an asymptomatic population with zero calcium score, the NNT with aspirin is approximately two thousand individuals to prevent one major cardiovascular event, while the number needed to harm (NNH) is 442 individuals, demonstrating a much greater risk in the prescription of aspirin than its benefits^15^. A cost-effectiveness analysis showed that the aspirin is only cost-effective in men with clinical risk in ten years greater than 10% and in women when the risk is > 15%^16^. In our study, aspirin was started in 5% of patients after a completely normal CCTA result, which probably would not be indicated, considering that the annual risk is < 0.1% of combined coronary events^9,16,17^. Conversely, we observed that 22% of patients with positive CCTA (obstructive or not) in our study started aspirin therapy.

The proportion of patients that initiated therapy with CLD has increased significantly as the severity of CAD increased (normal CCTA = 12%; non-obstructive CAD = 24%; CAD ≥ 50% = 43%), demonstrating that in the real world there is an agreement between therapy intensity and the severity of CAD lesions. There was no reduction in NLDLC in patients with normal CCTA, which occurred in patients with obstructive lesions ≥ 50%. There was a NLDLC decrease trend in patients with non-obstructive lesions, and these data are corroborated by other studies ^9,15,18,19^. Among the 42 patients with obstructive CAD ≥ 50%, 12 (29%) maintained the prescription of statin and/or aspirin and 24 (57%) started the use of the two drugs or added one of the two to previous therapy, after the CCTA result, corroborating the results of other published studies^19-23^.

A meta-analysis^24^ involving 170,000 patients showed that a 1-mmol/L decrease in LDL cholesterol was able to reduce the risk of cardiovascular events by 20%. We observed in this study that 28 (29%) showed LDL decrease > 1 mmol/L, which implies a reduction of cardiovascular risk in this group.

The reduction in all lipid fractions, except HDL, demonstrates not only the potent LDL-lowering effect of the CLD, as well as reductions more related to changes in dietary habits, as demonstrated by the reduction in triglycerides ^1,4,19^.

Of all patients considered at high risk by CCTA (lesion ≥ 50%), 32 (76%) reached the LDL target <100 mg/dL. This result contrasts with the data collected by Vacanti et al.^25^in a Brazilian population, in which it was found that only 30% of patients had LDL values within the Guideline’s targets.

The stratification of cardiovascular risk made by visualization of coronary atherosclerosis has previously demonstrated to have greater impact on medication adherence and change in clinical management than the risk stratification by clinical scores^20,21,26-29^. The results of this study indirectly corroborate that, showing patients with higher atherosclerotic disease severity have higher reduction in cholesterol levels.

Hulten et al.^26^ recently addressed the impact of CCTA findings on drug therapy in a study that evaluated 2,839 patients with a mean follow-up of 3.6 years, and found that after the CCTA examination there was an increase in the prescription of aspirin even in the group without CAD (10-46% vs. 17%-72% vs. 25%-89%, p = 0.001). This study also found statin prescription intensification after the CCTA results. After the CCTA results, 36% of patients without CAD were using CLD and 18% had been prescribed therapy intensification; in patients with non-obstructive lesions < 50%, 72% were using CLD and in 42%, prescription intensified occurred; in patients with lesions > 50% in stenosis, of the total 90% in use of statin, there was an intensification of 63%.

Among the assessed patients, two of them are especially illustrative for the analysis of the results. The first one, aged 54, had fatigue on moderate exertion, hypertension and did not use statins or aspirin. He had NLDLC of 201 mg/dL in the index visit. The CCTA disclosed 50% -70% lesion in the middle third of the Anterior Descending Artery (ADA). In the follow-up assessment, the patient was using aspirin and CLD and the NLDLC had decreased to 140 mg/dL. The second patient, aged 66 years, had atypical chest pain, diabetes, dyslipidemia, was hypertensive and sedentary, was using aspirin and CLD in the index visit had a NLDLC of 120 mg/dL. The CCTA disclosed a 50% -70% lesion in the proximal third of the ADA. At the second assessment, the NLDLC had decreased to 100 mg/dL with medication maintenance. The two patients were at high risk for cardiovascular events, but in the second case, there was not so marked decrease in NLDLC values, probably because the patient was already undergoing medical treatment and the medication was only optimized after CCTA the result.

### Study limitations

Some limitations should be considered in our study, which was observational and retrospective, performed at a single diagnostic imaging center, with two CTs of different manufacturers, with a relatively small sample. Because we selected patients with two sequential cholesterol measurements, there may have been some selection bias, preferably excluding patients that had not started treatment with CLD and therefore had no clinical need to collect a new blood sample for lipid level measurement. Therefore, the results are more relevant for the analysis of factors involved in the prescription of antiplatelet agents and CLD than for the lack of their indication.

## Conclusions

The severity of CAD positively correlated with the decrease in cholesterol levels and more frequent prescription of antiplatelet agents and CLD. These data suggest that the CCTA result can influence drug treatment approach in the short term in the real world.
